# Circulating Progenitor Cell Count for Cardiovascular Risk Stratification: A Pooled Analysis

**DOI:** 10.1371/journal.pone.0011488

**Published:** 2010-07-09

**Authors:** Gian Paolo Fadini, Shoichi Maruyama, Takenori Ozaki, Akihiko Taguchi, James Meigs, Stefanie Dimmeler, Andreas M. Zeiher, Saula de Kreutzenberg, Angelo Avogaro, Georg Nickenig, Caroline Schmidt-Lucke, Nikos Werner

**Affiliations:** 1 Department of Clinical and Experimental Medicine, University of Padova Medical School, Padova, Italy; 2 Department of Nephrology, Nagoya University Graduate School of Medicine, Nagoya, Japan; 3 Department of Cerebrovascular Disease, National Cardiovascular Center, Osaka, Japan; 4 Harvard Medical School and General Medicine Division, Massachusetts General Hospital, Boston, Massachusetts, United States of America; 5 Molecular Cardiology and Internal Medicine III, Wolfgang Goethe University, Frankfurt, Germany; 6 Department of Internal Medicine II, Division of Cardiology, Pneumology, and Angiology, University Hospital Bonn, Bonn, Germany; 7 Department of Cardiology and Pneumology, Charité, Universitätsmedizin Berlin, Campus Benjamin Franklin, Berlin, Germany; Innsbruck Medical University, Austria

## Abstract

**Background:**

Circulating progenitor cells (CPC) contribute to the homeostasis of the vessel wall, and a reduced CPC count predicts cardiovascular morbidity and mortality. We tested the hypothesis that CPC count improves cardiovascular risk stratification and that this is modulated by low-grade inflammation.

**Methodology/Principal Findings:**

We pooled data from 4 longitudinal studies, including a total of 1,057 patients having CPC determined and major adverse cardiovascular events (MACE) collected. We recorded cardiovascular risk factors and high-sensitive C-reactive protein (hsCRP) level. Risk estimates were derived from Cox proportional hazard analyses. CPC count and/or hsCRP level were added to a reference model including age, sex, cardiovascular risk factors, prevalent CVD, chronic renal failure (CRF) and medications. The sample was composed of high-risk individuals, as 76.3% had prevalent CVD and 31.6% had CRF. There were 331 (31.3%) incident MACE during an average 1.7±1.1 year follow-up time. CPC count was independently associated with incident MACE even after correction for hsCRP. According to C-statistics, models including CPC yielded a non-significant improvement in accuracy of MACE prediction. However, the integrated discrimination improvement index (IDI) showed better performance of models including CPC compared to the reference model and models including hsCRP in identifying MACE. CPC count also yielded significant net reclassification improvements (NRI) for CV death, non-fatal AMI and other CV events. The effect of CPC was independent of hsCRP, but there was a significant more-than-additive interaction between low CPC count and raised hsCRP level in predicting incident MACE.

**Conclusions/Significance:**

In high risk individuals, a reduced CPC count helps identifying more patients at higher risk of MACE over the short term, especially in combination with a raised hsCRP level.

## Introduction

Cardiovascular disease (CVD) is the leading cause of death in western countries. Thus, identification of patients at risk for future CVD must be pursued in order to implement preventive strategies. Traditional cardiovascular risk factors are commonly used for this purpose and many risk scores have been proposed based on various combinations of risk factors. However, a significant number of cardiovascular events still occur in subjects classified in the low or intermediate risk categories [1], thus reducing the chance to apply disease prevention in many subjects who would benefit from it. Identification of emerging risk factors and novel biomarkers of CVD has recently gained attention, in an attempt to improve the performance of risk prediction algorithms. A number of CVD biomarkers have been identified, many of which are independently associated with incident cardiovascular events in survival analyses [2]. However, the usefulness of testing biomarkers in the clinical setting has been questioned, because there is no definite evidence that biomarkers, alone or in combination, improve cardiovascular risk stratification and identification of patients at risk for future CVD. Indeed, it is increasingly recognized that basic association measures are insufficient to assess prognostic utility of biomarkers while newer methods, that assess how well biomarkers assign patients to clinical risk categories [3], yielded rather disappointing results [4], [5].

Inflammatory molecules are among the most extensively studied CVD biomarkers. For instance, a mildly raised C-reactive protein (CRP) reflects a condition of chronic low-grade inflammation that is considered one underlying cause of CVD development and progression [6]. However, inconsistency exists regarding the ability of CRP testing to improve risk assessment [7].

In the last decade, pathogenic models of CVD have moved to consider the role of circulating cells potentially involved in cardiovascular repair [8]. Endothelial progenitor cells (EPCs) are bone marrow-derived cells able to migrate into the bloodstream and participate in endothelial regeneration and angiogenesis [9], [10], [11]. Many animal models confirm the protective effects of EPCs on the cardiovascular system, and clinical studies show that low levels of circulating EPCs associate with prevalent and incident CVD [12], [13], [14]. Different phenotypes of circulating progenitor cells (CPC), including EPCs, are thus emerging as novel CVD biomarkers, which are also involved in disease pathogenesis [15]. In survival analyses of longitudinal studies, a reduced CPC count has been shown to independently predict cardiovascular events in patients with CVD [13], [16], chronic renal failure [17] or metabolic syndrome [18], but it is still not clear if CPC count is useful in the clinical setting for cardiovascular risk stratification. Re-analysis of individual data from relevant prospective studies of cardiovascular outcomes is emerging as a mean to address this uncertainty in a rapid and cost-effective manner [19].

This study, resulting from the collaboration of 4 independent research groups, tested the hypothesis that: i) adding CPC count to a standard risk model for cardiovascular risk stratification of high-risk individuals has a significant incremental predictive value; ii) the relationship between CPC and incident cardiovascular events is modified by inflammation and there is an interaction between CPC and CRP levels in cardiovascular event prediction.

## Methods

### Participants

This study was conceived as a post-hoc re-analysis of crude data from 4 previously published cohorts [13], [16], [17], [18]. The individual studies used for this pooled analysis were approved by the respective local Institutional Ethical committees (University of Saarland, J.W. Goethe University of Frankfurt, University Hospital of Padova and Nagoya Kyoritsu Hospital), and written informed consent was obtained from all subjects at time of the study. Investigators of each source study provided patients' data on the basis of an agreed protocol and data scheme. The following data were recorded for all patients: age, sex, smoking habit, presence of cardiovascular risk factors, chronic renal failure (CRF), prevalent CVD, and use of drugs. Twelve patients were excluded because of missing at least one the above-mentioned parameters. Shared definitions of cardiovascular risk factors were used: diabetes mellitus was defined by fasting plasma glucose ≥126 mg/dL or self-reported diabetes; smoking status was defined as habitual smoking of ≥1 cigarette per day; hypertension was defined as systolic blood pressure ≥140 mmHg or a diastolic blood pressure ≥90 mmHg, or the use of anti-hypertensive drugs; dyslipidemia was defined as either a total cholesterol concentration ≥200 mg/dL or a triglycerides concentration ≥200 mg/dl or a HDL cholesterol concentration of less than 40 mg/dl in men and 50 mg/dl in women or the use of statin/fibrates. CRF was defined as serum creatinine >1.3 mg/dL for at least 6 months or if the patient was on dialysis. CVD was defined as any of the following: a history of previous myocardial infarction or stable angina, a significant coronary artery diseases at angiography, peripheral arterial disease (claudication, rest pain or ischemic foot ulcers), cerebrovascular disease (a history of stroke or carotid atherosclerosis), presence of abdominal aortic aneurysm.

We also collected data on high sensitive C-reactive protein (hsCRP) concentrations, which were categorized as high and low according to an established cut-off (≤3.0 mg/L or >3.0 mg/L) [20]. hsCRP was measured using the turbidimetric method of Roche Diagnostics [13], [21] or Behring's ultrasensitive LatexCRP monotest [16], or the latex-enhanced high-sensitive CRP immunoassay (Nittobo Medical Co. Ltd) [22].

### Circulating progenitor cell count

CPC were defined as circulating CD34+KDR+ cells in 2 studies [13], [16], or as circulating CD34+ cells in the other 2 studies [17], [18]. Given the different definitions and measures of CPC, we adopted a strategy to render CPC count as much comparable as possible, by expressing CPC as belonging to a tertile of the normal distribution within each cohort. Thus, CPC count in the pooled sample could be reported as high (3^rd^ tertile), intermediate (2^nd^ tertile) or low (1^st^ tertile). A review of previous data suggest that CD34+ cell level is more stable over time than CD34+KDR+ cell level, which is more influenced by pharmacological treatment [23], [24].

### Follow-up and definition of the endpoint

In all source studies, follow-up was conducted by telephone contact, ambulatory visit or consultation of death registry. Potential events were verified by analysis of medical records, such as hospital charts and discharge letters. The main outcome measure of this pooled study was a modified definition of major adverse cardiovascular event (MACE). An incident MACE was recorded if the patient matched one of the following conditions during the follow-up period: cardiovascular (CV) death; non-fatal acute myocardial infarction (AMI); hospitalization for unstable angina or congestive heart failure (according to Framingham criteria [25]); coronary or peripheral revascularization procedure; angiographic evidence of restenosis after coronary revascularization; major amputation due to peripheral ischemia, stroke or transient ischemic attack. Event-free survival analyses were also performed separately for CV death, non-fatal AMI, non-fatal stroke and other CV events.

### Statistical methods

Continuous data were reported as mean ± standard error of the mean (SEM), and categorical data as percentage. Event-free survival was assessed with Cox proportional hazard analyses. Four different sets of variables were constructed, to be entered into 4 models, respectively. In model 1 (reference model), sex, age, cardiovascular risk factors, CRF, prevalent CVD and use of statins and ACE inhibitors/ARBs were forced into the model. This reference model was built to include all standard predictors of cardiovascular events that could be retrieved from all source studies. We included only those medications that were supposed to influence both outcome and CPC, to be controlled for. In model 2, variables of model 1 plus CPC were entered; in model 3, variables of model 1 plus hsCRP were entered; in model 4, variables of model 1 plus CPC and hsCRP were entered simultaneously. Estimated risk functions were calculated using beta coefficients from survival analyses and exponential transformation, similarly to what described for generating the Framingham risk equation. Risks estimated by Cox regressions were used to compare the performance of the 4 models. Average C-statistics was calculated as the area under ROC curve using either the logistic approach, which ignores time-to-event, or Chambless and Diao's method [26] and Harrell's method [27], which add time component to area under curve estimation. Confidence intervals for Ĉ were calculated based on Kendall's τ approximation as proposed by Pencina et al.[28]. P-values for comparison between Ĉ were computed from approximation to a normal distribution. Improvement in model performance with addition of CPC and/or hsCRP was also assessed by calculating the net reclassification improvement (NRI) with pre-specified tertile categories of risk and the integrated discrimination improvement (IDI), as previously described [3].

To explore the interaction between CPC and hsCRP levels in relation to incident MACE, we divided patients into 6 groups according to CPC tertiles and hsCRP<>3.0 mg/L. We then compared unadjusted event rates using χ^2^ and adjusted relative risks (RR) derived from Cox regression analysis of model 1 in these categories of subjects. Rothman's synergy index, a measure of interaction as departure from additivity, was calculated as previously described using adjusted RRs [29]. Confidence interval of synergy index was calculated as suggested by Zou [30]. SPSS versions 13.0 was used and statistical significance was accepted at p<0.05.

## Results

### Patients' characteristics

Clinical characteristics of the study patients are summarized in Table 1. The study sample was representative of a high risk population, as 76.3% of patients had CVD at baseline and 31.7% had chronic renal failure. This is in compliance with a relatively high incidence of MACE (331 events; 31.3% of subjects) over a relatively short follow-up time (1.7±1.1 years). Events were distributed as follows: 48 CV deaths, 19 non-fatal AMI, 19 non-fatal stroke, and 245 other CV events.

**Table 1:** Characteristics of study patients.

Characteristic	Value
Age (years, mean ± SEM)	63.1±0.4
Male gender (%)	64.2
Smoking (%)	24.3
Diabetes (%)	32.0
Hypertension (%)	70.7
Dyslipidemia (%)	56.8
hsCRP >3.0 mg/L (%)	39.1
Chronic renal failure/dialysis (%)	31.7
Prevalent CVD (%)	76.3
Statin use (%)	33.3
ACE inhibitor/ARB use (%)	51.4

### Survival analysis

**Figure 1:**
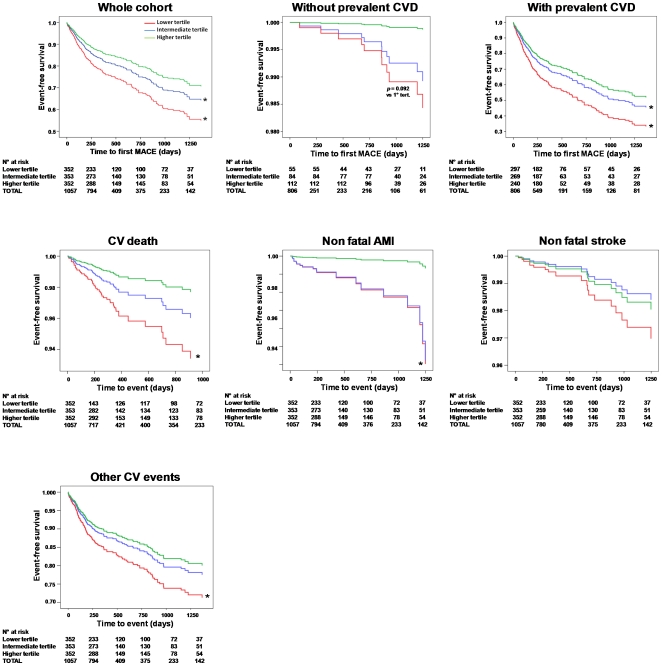
Kaplan-Meier Curves. Different curves are plotted for patients belonging to the different CPC tertiles in the whole cohort, and in groups of patients with or without prevalent CVD at baseline. Separate curves are also shown according to event type in whole cohort. Survival is corrected for confounders entered in model 4. *significantly different versus the higher CPC tertile group.

Cox proportional hazard analyses were performed to derive different prediction models (Table 2). In model 1 (reference model), hypertension, dyslipidemia, and prevalent CVD were significant predictors of incident MACE. Both low CPC and raised hsCRP (>3.0 mg/L), that were added respectively in models 2 and 3 were significant event predictors besides hypertension, dyslipidemia and CVD. CPC count was a significant predictor of incident MACE also in model 4, independently of hsCRP, dyslipidemia and CVD. Patients were then divided into 2 groups according to the presence/absence of prevalent CVD at baseline and model 4 was run for both: CPC tertile was a significant inverse event predictor in the CVD group, while there was a non-significant trend for a higher event rate with decreasing CPC tertile in the non-CVD group. Regarding event type, higher CPC tertile in model 4 was an independent inverse predictor of CV death (RR = 0.59; p = 0.007), non-fatal AMI (RR = 0.50; p = 0.037) and other CV events (RR = 0.81; p = 0.009), while it was not significantly associated with incident stroke/TIA (RR = 0.78; p = 0.404). Figure 1 shows Kaplan-Meier curves of incident events according to CPC tertiles (model 4) in the different groups.

**Table 2:** Results of the Cox hazard-proportional analyses.

Variable	Model 1 (reference)		Model 2 (+CPC)		Model 3 (+hsCRP)		Model 4 (+CPC+hsCRP)	
	RR	p	RR	p	RR	P	RR	p
**Male gender**	1.17	0.207	1.19	0.159	1.15	0.254	1.17	0.206
**Age (for 10 yrs)**	1.03	0.577	1.02	0.672	1.02	0.773	1.01	0.861
**Smoke**	0.94	0.668	0.92	0.551	0.93	0.611	0.92	0.512
**Diabetes**	1.18	0.151	1.14	0.266	1.18	0.154	1.13	0.290
**Hypertension**	1.45	0.022	1.38	0.046	1.45	0.023	1.36	0.061
**Dyslipidemia**	1.50	0.003	1.46	0.006	1.48	0.005	1.44	0.008
**Chronic renal failure**	0.98	0.894	0.97	0.814	0.96	0.782	0.95	0.708
**Prevalent CVD**	10.90	<0.001	10.48	<0.001	10.05	<0.001	9.57	<0.001
**Use of statin**	1.17	0.210	1.20	0.144	1.15	0.270	1.19	0.174
**Use of ACEI/ARBs**	0.96	0.767	0.97	0.817	0.98	0.838	0.98	0.900
**CPC tertiles**	-	-	0.77	<0.001	-	-	0.76	<0.001
**hsCRP>3.0 mg/L**	-	-	-	-	1.52	<0.001	1.57	<0.001

All explanatory variables were entered simultaneously in the model. CPC was entered as a continuous variable and relative risk (RR) expressed per tertile increase. RR for age is reported for each 10 yrs increase. ACEI, angiotensin converting enzyme inhibitors; ARB, angiotensin receptor blockers.

Linear risk functions were then calculated for each model using regression coefficients of survival analyses and exponential transformation, similarly to the equation used to derive the Framingham 10-year risk. Discrimination and performance of the risk estimates based on the 4 models were then assessed.

### Effects of CPC on discrimination of survival models

**Figure 2:**
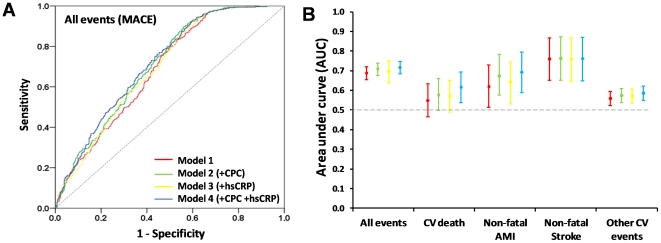
Discrimination analysis. Panel A shows ROC curves: logistic Ĉ is shown for each model. Panel B shows AUCs of logistic Ĉ with 95% confidence intervals (bars) according to event type and model 1 to 4.

Average C (Ĉ) was calculated using 3 methods. Logistic Ĉ, which ignores time-to-event, was not significantly increased in models 2, 3 and 4 as compared to model 1 (Figure 2A). Figure 2B shows that AUCs from logistic Ĉ increased not significantly also when CV death, non-fatal AMI and other CV events were considered separately. Similarly, Chambless and Diao's Ĉ, which adds time component to the area under ROC curve estimation, was not significantly higher when CPC count was entered in the model, with our without hsCRP, as compared to model 1. Harrell's Ĉ, which is independent of calibration, showed no significance discrimination improvement in model 2, 3 and 4, as well. As expected [3], Ĉ was highest with the logistic approach and lowest with Harrell's method for all models (Table 3). These results indicate that, on the basis of C-statistics, the addition of CPC did not significantly improve discrimination of the new survival model in comparison with a standard reference model.

**Table 3:** Performance of MACE prediction models using average C (Ĉ).

	Model 1 (reference)	Model 2 (+CPC)	Model 3 (+hsCRP)	Model 4 (+hsCRP+CPC)
**Logistic Ĉ**	0.687 (0.655–0.719)	0.707 (0.676–0.738)	0.695 (0.663–0.727)	0.716 (0.685–0.747)
**Chambless and Diao's Ĉ**	0.691 (0.642–0.731)	0.707 (0.663–0.750)	0.695 (0.651–0.739)	0.716 (0.673–0.759)
**Harrell's Ĉ**	0.631 (0.596–0.666)	0.635 (0.600–0.671)	0.644 (0.609–0.677)	0.648 (0.614–0.683)

95% confidence intervals reported in brackets.

### Effects of CPC on improvement in model performance

We then assessed whether the models including CPC with or without hsCRP yielded a better reclassification of patients in terms of MACE prediction. To this end, the NRI was calculated based on reclassification across tertiles of risk categories yielded by new models in comparison to the reference model. Movement of patients with incident MACE in higher risk categories and movement of patients without incident MACE in lower risk categories were considered as correct reclassifications. As shown in Table 4, in comparison to the reference model, inclusion of either CPC or hsCRP was not associated with a statistically significant NRI. Inclusion of both CPC and hsCRP in the model yielded better reclassification of 6.5%, but still was not statistically significant (p = 0.13). Re-analysis by event type indicated that inclusion of CPC measurement provided significant NRI for CV death (model 2 vs model 1: NRI = 18.6%, p = 0.034; model 4 vs model 1: NRI = 22.7%, p = 0.014), non-fatal AMI (model 2 vs model 1: NRI = 21.5%, p = 0.043), and other CV events (model 2 vs model 1: NRI = 6.5%, p = 0.015; model 4 vs model 1: NRI = 11.9%, p<0.001), but not for non-fatal stroke.

**Table 4:** Improvement of model performance.

	NRI	IDI
**Model 2 vs Model 1**	1.5% (p = 0.71)	0.017 (p = 0.0003)
**Model 3 vs Model 1**	−3.4% (p = 0.40)	0.011 (p = 0.013)
**Model 4 vs Model 1**	6.3% (p = 0.13)	0.029 (p<0.0001)
**Model 3 vs Model 2**	6.1% (p = 0.16)	−0.006 (p = 0.38)
**Model 4 vs Model 2**	5.1% (p = 0.17)	0.012 (p = 0.008)
**Model 4 vs Model 3**	10.0% (p = 0.008)	0.018 (p = 0.0002)

Net reclassification improvement (NRI) is reported as the net percentage of patients correctly reclassified by the new model across tertiles of MACE risk categories. The integrated discrimination improvement (IDI), which can be interpreted as a continuous version of NRI, is reported as absolute value.

Given that the NRI is highly dependent upon the pre-specified categories of risk, we also calculated the integrated discrimination improvement (IDI), which is a continuous assessment of reclassification improvement, not based on risk categories. IDI showed significant better discrimination by the models including CPC with or without hsCRP as compared to the reference model. Interestingly, there also was a significant IDI in the comparison of model 4 with models 2 and 3, suggesting that the inclusion of both CPC and hsCRP improved discrimination over the inclusion of either CPC alone or hsCRP alone. Then, an interaction between CPC and hsCRP was looked for.

### Interaction between CPC and hsCRP

**Figure 3:**
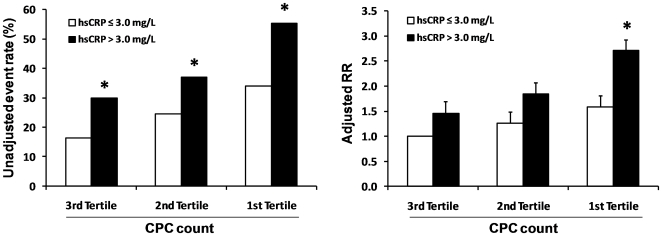
Interaction between CPC and hsCRP levels. Patients were divided into 6 groups according to CPC tertiles and high/low hsCRP. Left panel shows unadjusted events rates (* significantly different in χ^2^ analysis versus hsCRP≤3.0 mg/L). Right panel shows adjusted relative risks (RR) from model 1 (Bars  =  SE; * significantly different versus hsCRP≤3.0 mg/L).

Patients were divided into groups according to their concentration of hsCRP (<>3.0 mg/L) and their belonging tertile of CPC count. As shown in Figure 3, the risk of incident MACE across CPC tertiles was different in the high versus low hsCRP population: unadjusted event rates were significantly higher in patients with a hsCRP>3.0 mg/L across all CPC tertiles. After adjusting for age, sex, cardiovascular risk factors, CRF, prevalent CVD and medications (model 1), a high hsCRP was significantly associated with a higher relative risk (RR) of events in patients in the lowest CPC tertile. The slope of the relationship between CPC tertiles and RR of MACE was significantly higher in the high than in the low hsCRP group (8.81 [95% C.I. 8.12–9.51] versus 12.70 [95% C.I. 12.07–13.33]; p<0.001). This trend was suggestive of an interaction between CPC and hsCRP in relation to incident MACE. Rothman's synergy index, calculated as the excess risk in patients with both low CPC and high hsCRP divided by the sum of excess risk in patients either low CPC or high hsCRP, was significantly different from zero ( = 1.709/[0.450+0.589] = 1.64 [95% C.I. 1.04–2.60]; p = 0.032), indicating a more-than-additive interaction between CPC in the lower tertile and hsCRP>3.0 mg/L in determining incident MACE.

### Subsidiary analyses

**Figure 4:**
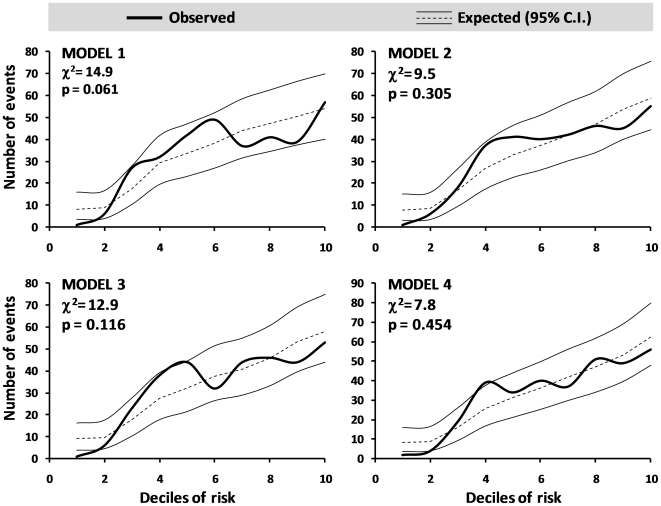
Calibration of predictive models. Deciles of risk were calculated for each model. Observed and expected even rates are plotted against deciles of risk. 95% confidence intervals (C.I.) for expected data according to the respective model were calculated according to the Poisson distribution. Results of the Hosmer-Lemeshow χ^2^ test is shown for each model.

Calibration analyses, performed using the Hosmer-Lemeshow test, indicated no significant differences between observed and expected event rates in all models and the χ^2^ value was lower in models including CPC (model 2 an 4). Accordingly, observed event rates across deciles of risk almost always fall within the 95% confidence interval of expected event rates (calculated according to the Poisson distribution), indicating good calibration (Figure 4).

Since the phenotype of CPC was inconsistent among studies (CD34+KDR+ in 2 studies and CD34+ in 2 studies), we performed distinct Cox regression analyses for subjects with CD34+ or CD34+KDR+ cell counts. We found that both CPC phenotypes were independent event predictors besides hsCRP in model 4 (not shown), re-assuring us on the possibility to merge together the cohorts. Further, when available data on CD34+KDR+ cells of the Italian cohort [18] were merged to data of the other 2 studies using CD34+KDR+ cell count (making a total of 842 patients with a homogenous CPC definition), CPC count was still an independent event predictor besides hsCRP in model 4 (not shown), but improvement in C-statistics was minimal (Logistic Ĉ: 0.730 [95% C.I.: 0.696–0.764] versus 0.729 [95% C.I.: 0.695–0.763]; p = 0.96. Chambless Ĉ: 0.718 [95% C.I.: 0.671–0.765] versus 0.715 [95% C.I.: 0.668–0.762]; p = 0.93). In 2 studies data on CD34+ and CD34+KDR+ cells could be retrieved. Thus, we compared categorization in tertiles using either CD34+ or CD34+KDR+ cells and found that 61.2% of patients were categorized in the same tertile by both definitions. Moreover, only 5% of these patients were categorized in the lowest tertile by one definition and in the higher tertile by the other definition, suggesting a good correspondence between CPC categorization in pooled cohorts.

## Discussion

This pooled analysis represents the first attempt to determine the ability of CPC count to improve cardiovascular risk stratification. Based on C-statistics, inclusion of CPC in the risk equation provided limited and non-significant improvement over and beyond a standard model based on classic risk factors. However, a less restrictive metric (the IDI) showed that the model including CPC outperformed the reference model in terms of accuracy of even prediction, independently and beyond the effect of hsCRP inclusion.

In each of the cohorts that compose the present study population, CPC count was a significant independent predictor of cardiovascular events [13], [16], [17], [18], but none of the source studies were well-powered to perform analysis of discrimination improvement. Indeed, large clinical studies on CPC are not available, because multicenter projects are hampered by the lack of standardized methods for CPC quantification, and because fresh blood samples for CPC determination must be processed within a few hours, thus limiting the possibility to analyze stored samples [31]. We tried to overcome these limitations by pooling crude data from distinct yet similar studies. Our results show that a low CPC count helps in identifying more patients at risk for future MACE, for the first time providing some evidence in support of a potential application of CPC count for cardiovascular risk stratification in the clinical practice. CPC are protective against the onset of CVD because they are involved in maintenance of a healthy endothelial layer, by means of promoting re-endothelialization of injured arteries [9]. Further, CPC are also protective against CVD progression as they promote compensatory angiogenesis in ischemic syndromes, thus limiting the extent of residual ischemia [11]. Therefore, it is expected that a paucity of these cells predispose to CVD onset or progression. Indeed, a reduced CPC count is linearly associated with severity of CVD involvement [12]. Furthermore, low CPC were found to predict incident events suggestive of CVD onset or progression in survival analyses of different cohorts of patients [13], [16], [17], [18]. Thus, CPC count is revealing as a novel prototype of surrogate biomarkers for cardiovascular risk, supported by both pathophysiological and epidemiological evidence. In the present study, we addressed the next important step in the evaluation pipeline of a putative biomarker, that is the incremental value in quantitative risk assessment over traditional risk factors [3]. Studying a high-risk population, we first confirm that CPC count is independently associated with incident events, and then looked at reclassification improvement yielded by addition of CPC measure beyond traditional demographics and risk factors. To this end, we used metrics specifically designed to assess the clinical utility of one or more biomarker(s) under scrutiny. Addition of CPC to a risk model built on conventional risk factors had marginal and non-significant effects on C statistics calculated using both the logistic method and methods that take into account time to event. This is not surprising, because C statistic is poorly sensitive to small changes in predictive accuracy, such that even established risk factors could be discarded as non-significant is some circumstances [32]. Indeed, it is very uncommon that a single surrogate biomarker improves C statistics when added to a well-fitted reference model; notably, in previous studies, even combinations of several biomarkers yielded modest changes in Ĉ when added to a standard risk assessment [5], [33]. Given the limitations of C statistics, we also calculated the IDI, a newer metric that improves when novel markers correctly assign individuals to higher or lower probabilities of having events. The IDI for MACE prediction improved significantly when either CPC or hsCRP were added to the reference model, and improved further when both were added together (Table 4). The NRI, a discrete version of IDI based on upward or downward movement across pre-specified risk categories, was significant for CV death, non-fatal AMI and other CV risk, but not for the combined MACE. We used risk tertiles to calculate the NRI given the impossibility to translate risk estimates in the present population into the clinically-relevant standard 10-year risk estimate. This might have affected results, since the NRI is highly sensitive to pre-specified categories.

Cumulatively, our data suggest that CPC measure may add incremental predictive value to standard risk assessment and that this effect might be modulated by hsCRP levels. Accordingly, we found a significant interaction between low CPC and high hsCRP levels in predicting incident events. After statistical adjustment, the excess risk of MACE in patients with both CPC in the lower tertile and hsCRP>3.0 mg/L was higher than the sum of excess risks in patients with either low CPC or high hsCRP, indicating a more-than-additive interaction between the two risk biomarkers in determining incident MACE. Biologically, this observation suggests that reduced vascular repair and inflammation are two distinct pathways of cardiovascular disease that synergize to increase the likelihood of adverse outcomes.

### Limitations

This study has limitations inherent to the pooling of data coming from 4 different cohorts. First, the definition of CPC in the source studies was different. The exact definition and cellular progeny of circulating (endothelial) progenitor cells is debated [34]. In this pooled analysis, by transforming CPC counts into tertiles, we could make data comparable and poolable, but potential biological differences between CD34+ cells (measured in 2 studies [17], [18]; n = 430) and CD34+KDR+ cells (measured in the other 2 studies [13], [16]; n = 627) might confound results. There is evidence that CD34+ and CD34+KDR+ cell counts are correlated each other and are subjected to consistent variations [35], but the CD34+ cells form a more generic population of progenitor cells, while CD34+KDR+ cells are primed to the endothelial lineage and can be considered EPC [31], [35]. Thus, future studies should focus on a single CPC phenotype, but our separated analyses for CD34+ and CD34+KDR+ cells showed consistent results, suggesting that there is no definite evidence that one phenotype is superior to the other(s) in terms of risk prediction. Our analyses are limited by the need to categorize CPC count to pool together the source studies; assessment of this surrogate biomarker along the continuous scale may provide better results and may offer the opportunity to define cutoffs. A second limitation is that methods for hsCRP measurement were not standardized among centers, and we simply could categorize hsCRP levels as high or low according to the standard 3.0 mg/L cutoff. Third, the original populations of patients are heterogeneous and the pooled cohort is mainly composed of high risk individuals in primary and secondary prevention. It is generally agreed that biomarkers perform better in high-risk than in low-risk populations [36] and, in the present study, more significant results were obtained in patients with baseline CVD. In addition, even if we tried to harmonize the endpoint by using a modified definition of MACE, event adjudication was not centralized.

### Future directions

Results of the present study need to be replicated in a more homogenous group of patients, yet large enough to allow statistical power in the analysis of discrimination improvement. Finally, to establish a definite causal link between reduced CPC and CVD onset or progression, studies with a pathophysiology-focused design are needed, such as mendelian randomization studies and/or biomarker-guided targeted treatment studies [37], [38]. Mendelian randomization studies could address polymorphisms in the *cd34* gene itself [39] or in the *cxcl12* gene, encoding the progenitor cell-regulating chemokine SDF-1α [40]. Interestingly, CPC levels are also potentially modifiable and amenable to pharmacological and non-pharmacological interventions. Many drugs currently used in the treatment of CVD, including statins and RAS blockers, have been shown to favorably modulate CPC [9], [41], [42]. Lifestyle interventions, such as diet [43], weight loss [44], exercise [45], and smoke cessation [46], have beneficial effects on CPC, as well. Therefore, besides being a pathogenetic actor, a disease biomarker and a prognostic indicator, CPC also appear to be a potential therapeutic target. It remains to be determined to what extent a therapeutic increase in CPC will translate into an improvement of event-free survival.

### Conclusions

Our data confirm that low CPC counts predicts adverse cardiovascular outcomes independently of chronic low grade inflammation, but synergistically with raised hsCRP levels. Analysis of this pooled cohort also supports the potential use of CPC count in cardiovascular risk stratification of high-risk individuals, especially in combination with the measure of hsCRP. A simplified CPC assessment by isolated CD34 expression analysis may be a simple and cheap way of measuring this new surrogate CV risk biomarker. Larger epidemiological and intervention studies are needed to understand the causal relationships between low CPC and CVD as well as the potential therapeutic implications.
